# MPEP Attenuates Intrahepatic Fat Accumulation in Obese Mice

**DOI:** 10.3390/ijms24076076

**Published:** 2023-03-23

**Authors:** Andrea Ferrigno, Marta Cagna, Oriana Bosco, Michelangelo Trucchi, Clarissa Berardo, Ferdinando Nicoletti, Mariapia Vairetti, Laura G. Di Pasqua

**Affiliations:** 1 Unit of Cellular and Molecular Pharmacology and Toxicology, Department of Internal Medicine and Therapeutics, University of Pavia, 27100 Pavia, Italy; 2 Interuniversity Center for the Promotion of the 3Rs Principles in Teaching and Research (Centro 3R), 56122 Pisa, Italy; 3 Department of Biomedical and Clinical Science, University of Milano, 20157 Milano, Italy; 4 Department of Physiology and Pharmacology, Sapienza University, 00185 Rome, Italy; 5 IRCCS Neuromed, 86077 Pozzilli, Italy

**Keywords:** MPEP, NAFLD, NASH, liver, high-fat diet, *ob/ob* mice, mGluR5

## Abstract

The blockade of metabotropic glutamate receptor type 5 (mGluR5) was previously found to reduce fat accumulation in HEPG2 cells. Here, we evaluated the effects of mGluR5 blockade in a mouse model of steatosis. Male *ob/ob* mice fed a high-fat diet were treated with MPEP or vehicle. After 7 weeks, liver biopsies were collected, and nuclei were isolated from fresh tissue. Lipid droplet area and collagen deposition were evaluated on tissue slices; total lipids, lipid peroxidation, and ROS were evaluated on tissue homogenates; PPARα, SREBP-1, mTOR, and NF-κB were assayed on isolated nuclei by Western Blot. Target genes of the above-mentioned factors were assayed by RT-PCR. Reduced steatosis and hepatocyte ballooning were observed in the MPEP group with respect to the vehicle group. Concomitantly, increased nuclear PPARα and reduced nuclear SREBP-1 levels were observed in the MPEP group. Similar trends were obtained in target genes of PPARα and SREBP-1, Acox1 and Acc1, respectively. MPEP administration also reduced oxidative stress and NF-κB activation, probably via NF-κB inhibition. Levels of common markers of inflammation (Il-6, Il1β and Tnf-α) and oxidative stress (Nrf2) were significantly reduced. mTOR, as well as collagen deposition, were unchanged. Concluding, MPEP, a selective mGluR5 negative allosteric modulator, reduces both fat accumulation and oxidative stress in a 7-week murine model of steatosis. Although underlying mechanisms need to be further investigated, this is the first in vivo study showing the beneficial effects of MPEP in a murine model of steatosis.

## 1. Introduction

Non-alcoholic fatty liver disease (NAFLD), which embraces both simple steatosis and non-alcoholic steatohepatitis (NASH), is considered the hepatic expression of the metabolic syndrome, which, in turn, comprises a wide spectrum of metabolic abnormalities, such as dyslipidemia, hypertension, insulin resistance, and diabetes [1]. Recently, NAFLD has been found to affect more than 25% of the general population worldwide [2], rising up to 75% in obese or diabetic people, even though the progression to NASH occurs in a limited portion of subjects [3].

The pathogenesis of NAFLD is considered a multifactorial process, as currently theorized in the Multiple-hit Hypothesis [4]. In fact, a wide range of parallel events are involved in the manifestation and progression of NAFLD, including nutritional habits [5], insulin resistance [6], altered gut microbiota [7], mitochondrial dysfunction [8], endoplasmic reticulum stress [9], activation of inflammatory pathways [10], and genetic and epigenetic factors [11]. A small percentage of cases, around 2%, are related to drug administration and classified as drug-induced steatohepatitis (DISH) [12].

To date, no drug has been specifically developed for the treatment of NAFLD. The antidiabetic drug pioglitazone is currently recommended as first-line off-label treatment of NAFLD only when chronic inflammation and fibrosis (NASH) are present; a safer but possibly less-effective option is Vitamin E; statins, when concomitantly used to reduce LDL-cholesterol and cardiovascular risk, have been found to represent no additional benefit or harm on liver disease [13]. In the absence of NASH, pharmacotherapy is not recommended; patients should be advised to have a more healthy diet and physical exercise [13].

In 2000, Storto and colleagues demonstrated the presence of metabotropic glutamate receptor (mGluR)5 in primary rat hepatocytes and HEPG2 cells using Western Blot, immunohistochemistry, and RT-PCR analysis [14]. The role of mGluR5 in liver injury was also investigated: mGluR5 agonists (1S,3R)-1-aminocyclopentane-1,3-dicarboxylic acid (ACPD) and quisqualate exacerbated ischemic damage in primary hepatocytes, while mGluR5 antagonist 2-methyl-6-(phenylethynyl)pyridine (MPEP), as well as mGluR5 gene depletion, protected from hypoxic injury [14,15]. In addition, MPEP administration was also found to reduce acetaminophen-induced liver toxicity in rats; in this work, the improvement of oxidative stress was not matched by an increase in GSH stores; on the contrary, a reduced iNOS activity in liver homogenates was demonstrated, suggesting that MPEP did not act by scavenging the toxic acetaminophen metabolite but by modulating inflammatory pathways [16]. MPEP-induced protection from hepatic ischemic injury was then confirmed in ex vivo models of hepatic cold and warm ischemia [17] and in an ex-vivo model of organ preservation with livers obtained from donors after cardiac death [18]. In both papers, MPEP treatment was associated with a reduced expression of TNF-α and iNOS and reduced apoptosis. Finally, MPEP was found to protect HEPG2 cells in an in vitro model of simple steatosis; but, interestingly, in addition to the expected negative modulation of inflammatory factors, MPEP administration was found to inhibit fat accumulation in HEPG2 cells by the modulation of sterol regulatory element-binding protein (SREBP)-1 and peroxisome proliferator-activated receptor (PPAR)-α [19]. SREBP-1 and PPARα are transcription factors relevant in hepatic lipid regulation, with SREBP-1 controlling cholesterol and lipid biosynthesis and PPARα being a key regulator of peroxisomal, mitochondrial, and microsomal fatty acid oxidation; in fact, ovariectomy depleted estrogen-induced protection from steatosis in female mice by reversing SREBP-1 and PPARα activation states [20].

The mechanism underlying the increase in mGluR5 signaling in various models of hepatic injury has not been completely elucidated yet; however, an increase in glutamate release was observed in isolated rat hepatocytes under hypoxia [14] and in a rat portal vein ligation model [21]. Recently, increased glutamate secretion was also found in a model of alcohol-induced steatosis; the consequential increase in mGluR5 signaling on hepatic stellate cells (HSC) resulted in abnormal fat accumulation in hepatocytes [22] and collagen deposition [23]. No study is currently available on the effect of MPEP in an in vivo model of NAFLD, so we evaluated the effects of MPEP administration in obese (*ob/ob*) mice fed with a high-fat diet for 7 weeks. Based on previous results, we evaluated lipid accumulation and oxidative stress, as well as the activation state of nuclear factors involved in both inflammatory pathways and lipid metabolism.

## 2. Results

In this work, we used *Lep^ob/ob^* (*ob/ob*) mice fed with a high-fat diet (HFD) for 7 weeks to induce NAFLD. In *ob/ob* mice, a spontaneous mutation in the leptin gene causes a lack of leptin in white adipose tissue, resulting in hyperphagic behavior, obesity, and inactivity. In animals older than 3–4 weeks, an altered metabolic profile is observed, leading to hepatocyte lipid accumulation, lipotoxicity, and lipoapoptosis. Differently from the methionine-choline deficient (MCD) model, *ob/ob* mice fed with a HFD also develop insulin resistance and hyperlipidemia and are considered more suitable than MCD mice for the study of metabolic syndrome [1,24].

### 2.1. Model Characterization and Liver Injury Evaluation

#### MPEP Reduces Body and Liver Weight

*Ob/ob* mice were fed with HFD for 7 weeks, concomitantly with intraperitoneal (IP) administration of vehicle (HFD group) or 20 mg/Kg MPEP (HFD + MPEP group). As reported in the literature, the administration of MPEP in a dose range between 0.05 mg/Kg and 30 mg/Kg produces a specific response in vivo [25]. The dosage was also determined based on a previous study in which the administration of 20 mg/Kg MPEP protected mice against acetaminophen hepatotoxicity [16]. At the end of the treatment, mice were anesthetized and weighed before blood and tissue collections for biochemical analyses. Data about body and liver weight at the time of sacrifice, as well as data about liver enzymes and plasma levels of glucose, are reported in Table 1. A significant reduction in both body and liver weight was found in the MPEP-treated group when compared with vehicle-treated mice. No significant differences were observed between the two groups in transaminase release as well as in plasma glucose levels.

### 2.2. MPEP Reduces Macrosteatosis, Hepatic Lipid Accumulation and Serum Triglycerides

**Figure 1:**
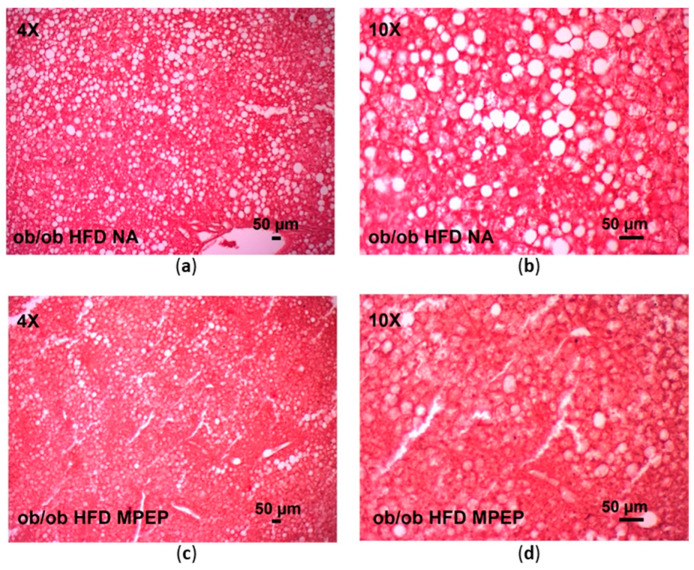
Hematoxylin-eosin staining of liver sections from obese mice after 7 weeks of HFD administration. (**a**,**b**) Obese mice receiving daily IP natrosol as vehicle; objective: 4× and 10×, respectively. (**c**,**d**) Obese mice receiving daily IP MPEP (20 mg/Kg); objective: 4× and 10×, respectively. HFD: high-fat diet; NA: natrosol. Scale bar: 50 μm.

After 7 weeks of HFD administration, livers from *ob/ob* mice receiving IP vehicle (HFD group) showed diffuse hepatocyte ballooning, overall lipid accumulation, and spotty focal necrosis (Figure 1a,b). Livers from *ob/ob* mice treated with daily IP MPEP 20 mg/Kg (HFD + MPEP group) had reduced hepatocyte ballooning when compared with the HFD group (Figure 1c,d).

**Figure 2:**
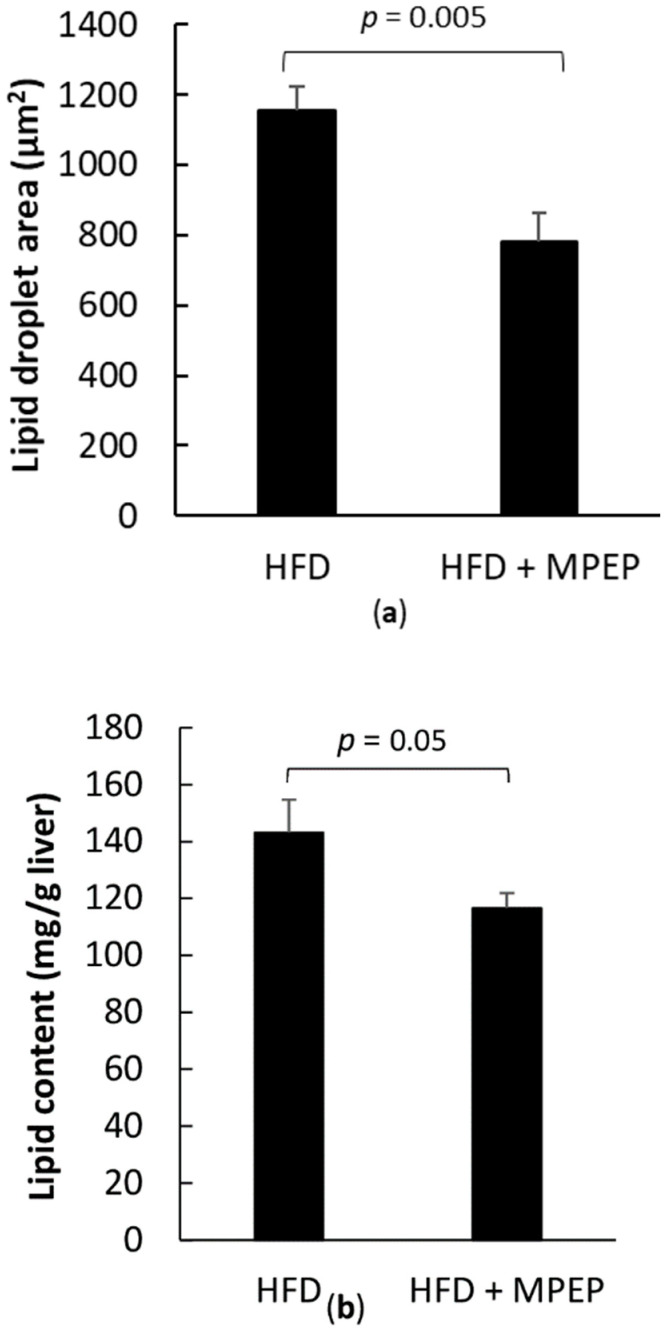
Lipid droplet area and total lipid content in obese mice. (**a**) Lipid droplet area was measured on H & E stained liver sections using ImageJ; (**b**) Total lipid content was obtained by fluorimetric measurements of Nile Red levels in lipid extracts. HFD: high-fat diet + vehicle group; HFD + MPEP: high-fat diet + 20 mg/Kg MPEP group. Results are mean ± SE of 6 individual determinations.

To quantify the MPEP-induced reduction in lipid accumulation, lipid droplet area was evaluated in Hematoxylin/Eosin stained liver sections using ImageJ software; in addition, total lipid content was assayed on tissue samples with the fluorescent dye Nile Red. A significant decrease in lipid droplet area was found in mice treated with MPEP (Figure 2a; *p* = 0.005); a significant reduction of total lipids was also found in liver lipid extracts from the MPEP group (Figure 2b; *p* = 0.05). 

**Figure 3:**
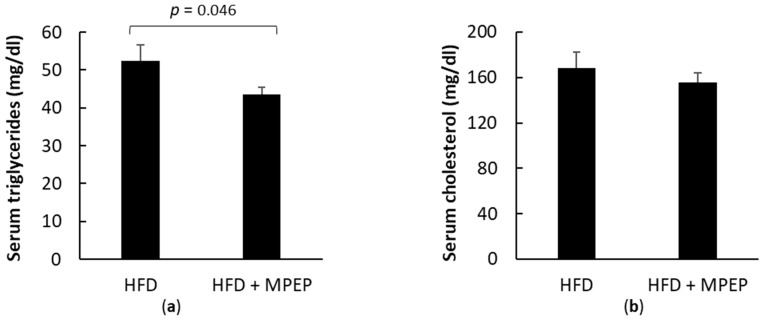
Plasma triglycerides and cholesterol in obese mice. (**a**) Triglycerides in plasma from obese mice after 7 weeks of HFD administration treated with MPEP 20 mg/Kg or vehicle; (**b**) Cholesterol in plasma from obese mice after 7 weeks of HFD administration treated with MPEP 20 mg/Kg or vehicle. HFD: high-fat diet + vehicle group; HFD + MPEP: high-fat diet + 20 mg/Kg MPEP group. Results are mean ± SE of 6 individual determinations.

Cholesterol and triglyceride levels were assayed in plasma. A significant decrease in serum triglycerides was observed in the HFD + MPEP group in comparison with the HFD vehicle group (Figure 3a; *p* = 0.046); no difference was observed in cholesterol levels (Figure 3b). These data show that MPEP daily administration is associated with reduced hepatic lipid accumulation and hepatocyte ballooning and reduced plasma triglycerides in *ob/ob* mice fed with a HFD.

### 2.3. MPEP Modulates Lipid Metabolism through SREBP-1 and PPARα in an mTOR-Independent Way

**Figure 4:**
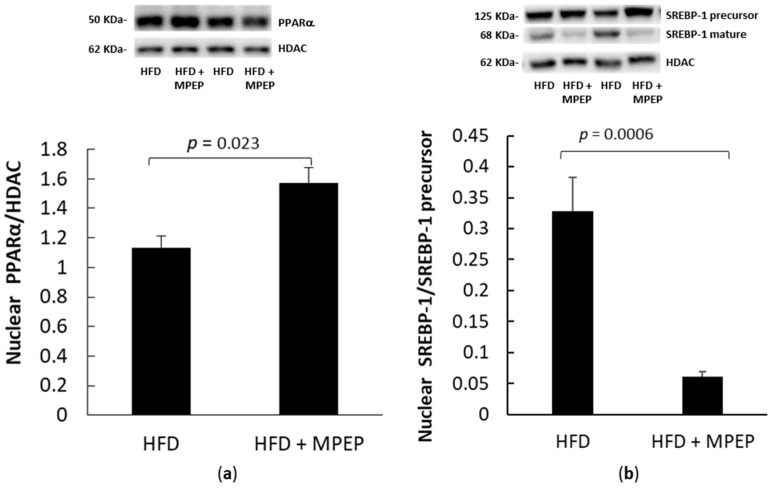
PPARα and SREBP-1 in obese mice. (**a**) PPARα in liver nuclear extracts from obese mice after 7 weeks of HFD administration, treated with MPEP 20 mg/Kg or vehicle; (**b**) mature SREBP-1/SREBP-1 precursor ratio in nuclear extracts from obese mice after 7 weeks of HFD administration, treated with MPEP 20 mg/Kg or vehicle. HFD: high-fat diet + vehicle group; HFD + MPEP: high-fat diet + 20 mg/Kg MPEP group. Results are mean ± SE of 6 individual determinations.

PPARα and SREBP-1 are pivotal regulators of lipid metabolism; in fact, PPARα promotes peroxisomal, mitochondrial, and microsomal fatty acid oxidation, and SREBP-1 activates cholesterol and lipid biosynthesis [26]. The activation levels of PPARα and SREBP-1 in nuclear extracts from *ob/ob* mice after 7-week HFD were evaluated to see whether MPEP daily administration affected lipid metabolism. In the MPEP group, a significant increase in the translocation of PPARα in nuclear liver extracts was found (Figure 4a, *p* = 0.023), concomitantly with a significant decrease in SREBP-1 activation (Figure 4b, *p* = 0.0006). Since the blockade of the mGluR5 receptor with MPEP is associated with a strong reduction of SREBP-1 activity and an increase in PPARα activity, these findings suggest that the mGluR5 receptor may be involved in the hepatic lipid accumulation observed in *ob/ob* mice.

PPARα downstream genes acyl-Coenzyme A oxidase 1, palmitoyl (Acox1), carnitine palmitoyltransferase 1a, liver isoform (Cpt1a), and SREBP-1 downstream genes acetyl-Coenzyme A carboxylase alpha (Acc1) and fatty acid synthase (Fasn) were also taken into account. RT-PCR analysis in hepatic tissue from *ob/ob* mice after 7-week HFD and treated with MPEP (20 mg/Kg) or vehicle was performed to investigate any changes in the above-mentioned genes. 

**Figure 5:**
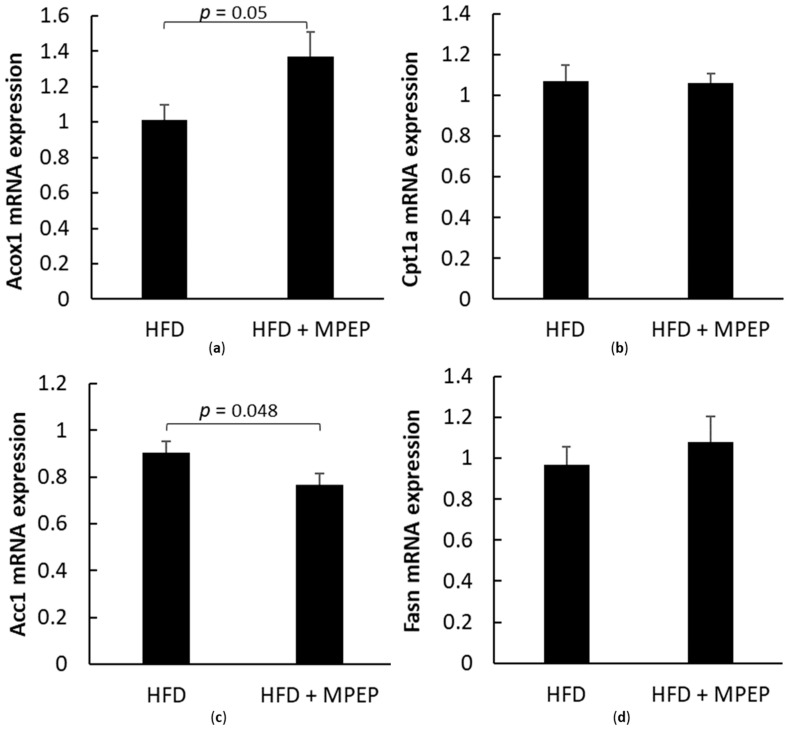
PPARα and SREBP-1 target genes in obese mice. (**a**) PPARα target gene Acox1 mRNA expression in liver from obese mice after 7 weeks of HFD administration, treated with MPEP 20 mg/Kg or vehicle; (**b**) Cpt1a mRNA expression in liver from obese mice after 7 weeks of HFD ad-ministration, treated with MPEP 20 mg/Kg or vehicle; (**c**) Acc1 mRNA expression in liver from obese mice after 7 weeks of HFD administration, treated with MPEP 20 mg/Kg or vehicle; (**d**) Fasn mRNA expression in liver from obese mice after 7 weeks of HFD administration, treated with MPEP 20 mg/Kg or vehicle. HFD: high-fat diet + vehicle group; HFD + MPEP: high-fat diet + 20 mg/Kg MPEP group. Results are mean ± SE of 6 individual determinations.

A significant increase was found in PPARα target gene Acox1 mRNA expression after MPEP administration (Figure 5a, *p* = 0.05), while no difference in Cpt1a transcript was observed (Figure 5b). The increase in Acox1 mRNA expression in the MPEP-treated group supports the hypothesis about the ability of MPEP to improve peroxisomal β-oxidation through PPARα activation.

Acc1 and Fasn are both target genes of SREBP-1, the lipogenic factor investigated in the present work. The mRNA expression of the Acc1 gene was significantly decreased after MPEP administration (Figure 5c, *p* = 0.048), in accordance with the observed reduction of SREBP-1 protein expression. Regarding the mRNA expression of Fasn, no differences were found between the two considered groups (Figure 5d).

**Figure 6:**
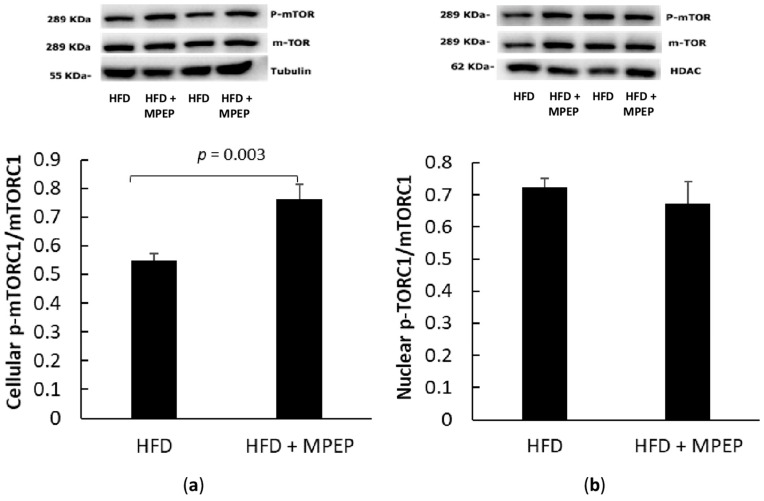
Activation of cellular and nuclear mTOR in obese mice. (**a**) activated mTOR in liver homogenates from *ob/ob* mice after 7 weeks of HFD administration and treatment with MPEP 20 mg/Kg or vehicle; (**b**) activated mTOR in nuclear extracts from *ob/ob* mice after 7 weeks of HFD administration, treated with MPEP 20 mg/Kg or vehicle. HFD: high-fat diet + vehicle group; HFD + MPEP: high-fat diet + 20 mg/Kg MPEP group. Results are mean ± SE of 6 individual determinations.

The mechanistic target of rapamycin (mTOR) is considered the central node of a network involved in NAFLD and NAFLD-associated hepatocellular carcinoma development. In particular, mTOR has been found to regulate lipid metabolism via SREBPs, insulin resistance, oxidative stress, intestinal microbiota, autophagy, inflammation, genetic polymorphisms, and epigenetics in NAFLD [26]. In most cases, the activation of the lipogenic SREBP seems to be mTOR-dependent. In this work, the mTOR phosphorylation site Ser2448, which produces the activation of the mTORC1 complex, was taken into account. The same phosphorylation site is widely investigated in the literature to observe mTORC1 activation in NAFLD models [27,28], and HFD administration generally results in a significant activation of Akt and further increased mTOR phosphorylation at Ser2448 [28]. Surprisingly, we found that nuclear mTOR was not altered by MPEP administration, suggesting that the consistent reduction in SREBP-1 activity observed in the HFD + MPEP group is not mediated by mTOR inhibition (Figure 6a,b).

**Figure 7:**
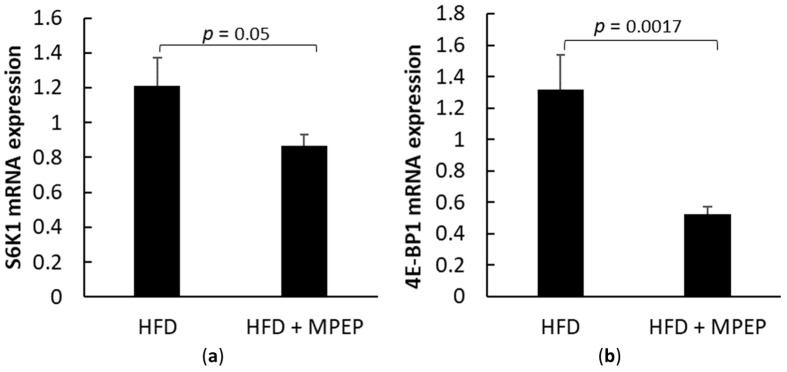
mTORC1 target genes in obese mice. (**a**) mTORC1 target gene S6K1 mRNA expression in liver from obese mice after 7 weeks of HFD administration, treated with MPEP 20 mg/Kg or vehicle; (**b**) 4E-BP1 mRNA expression in liver from obese mice after 7 weeks of HFD ad-ministration, treated with MPEP 20 mg/Kg or vehicle. Results are mean ± SE of 6 individual determinations.2.4. MPEP ameliorates oxidative stress and reduces the activation of pro-inflammatory NF-κB.

Ribosomal protein S6 kinase, polypeptide 1 (S6K1), and eukaryotic translation initiation factor 4E binding protein 1 (4E-BP1) are target genes of mTORC1: RT-PCR analysis in hepatic tissue from *ob/ob* mice after 7-week HFD treated with MPEP (20 mg/Kg) or vehicle was performed to investigate any changes in mTORC1 downstream genes. A significant decrease in the mRNA expression of both considered genes, S6K1 (Figure 7a, *p* = 0.05) and 4E-BP1 (Figure 7b, *p* = 0.0017) respectively, was found in the MPEP-treated group when compared with the vehicle-treated group. These results are apparently in contrast with the activation of mTORC1 in MPEP treated group shown in Figure 6; however, data from literature demonstrate that these two genes can be regulated independently of mTOR. In particular, S6K1 regulation can be attributed to the protein kinase RSK in response to growth factors and stress signals [29]. Literature data also show also mTOR-independent regulation of 4E-BP1 occurs in glioma cells [30], myoblasts [31], and acute myeloid leukemia [32]. This situation is in line with the reduction of S6K1 and 4E-BP1 mRNA expression found in this work in MPEP treated group.

**Figure 8:**
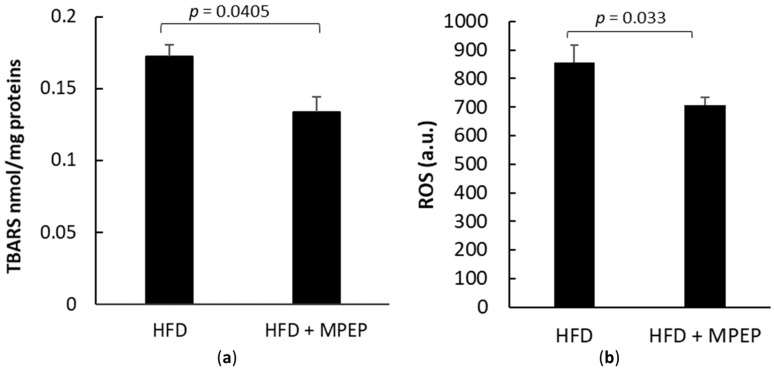
TBARS and ROS in obese mice. (**a**) TBARS in liver homogenates from obese mice after 7 weeks of HFD administration, treated with MPEP 20 mg/Kg or vehicle; (**b**) ROS in liver homogenates from obese mice after 7 weeks of HFD administration treated with MPEP 20 mg/Kg or vehicle. HFD: high-fat diet + vehicle group; HFD + MPEP: high-fat diet + 20 mg/Kg MPEP group. Results are mean ± SE of 6 individual determinations.

Lipid-induced over-production of reactive oxygen species (ROS) was traditionally considered the second hit in NAFLD pathogenesis [33] and is currently believed to be one of the concurring causes of NAFLD pathogenesis and progression [4]. During NAFLD development, ROS production worsens the scenario because it contributes to various other insults, such as cytokine-mediated inflammation, free fatty acid oxidation, apoptosis, necroinflammation, and fibrosis [33]. In this work, ROS were assayed using the fluorescent probe dihydro-dichlorofluorescein (DCFH). Thiobarbituric acid-reactive substance (TBARS) assay was used as an index of oxidative stress. In the MPEP group, a significant reduction of both TBARS and ROS was found, suggesting that daily MPEP of 20 mg/Kg reduces ROS production and, in the long run, oxidative stress caused by HFD (Figure 8a,b). 

**Figure 9:**
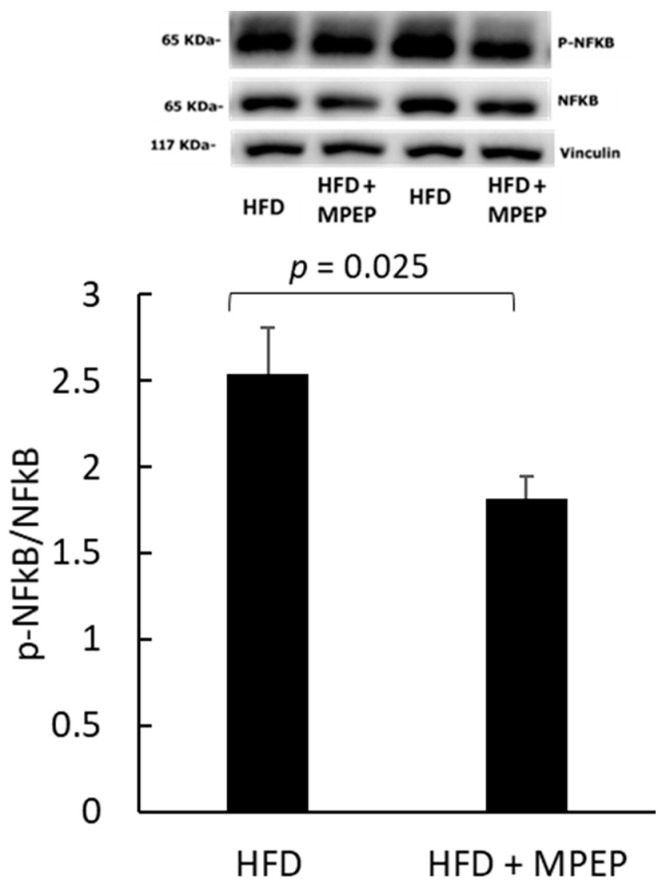
NF-κB activation in obese mice. NF-κB in liver homogenates from obese mice after 7 weeks of HFD administration, treated with MPEP 20 mg/Kg or vehicle. Results are mean ± SE of 6 individual determinations.

To investigate the possible causes of MPEP-induced reduction in oxidative stress, the activation state of the nuclear factor kappa-light chain enhancer of activated B cells (NF-κB) was also evaluated. In fact, studies have demonstrated that, in NAFLD, the inflammatory response is mediated by NF-κB via miR-125b, a miRNA highly expressed in this condition [34]. In this study, we found a significantly lower activation level of NF-κB in the MPEP group (Figure 9).

**Figure 10:**
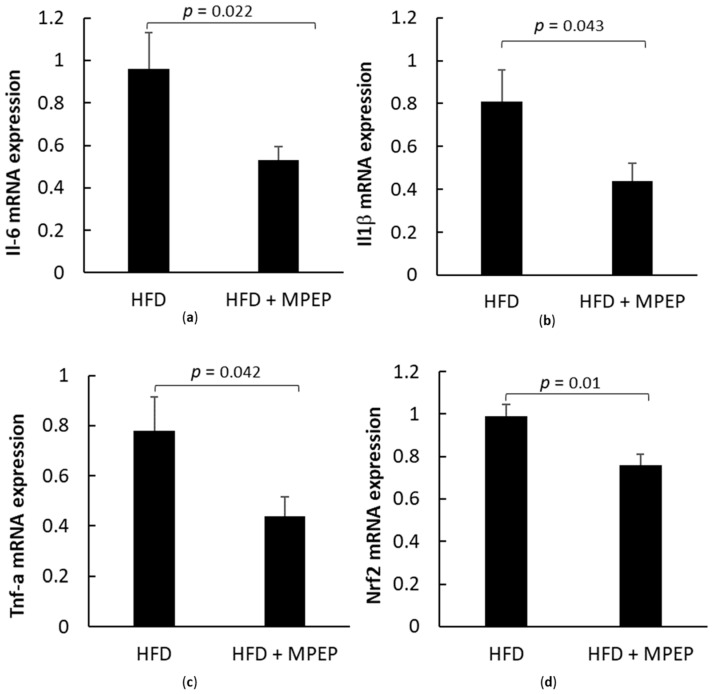
Inflammation and oxidative stress markers in obese mice. (**a**) Il-6 mRNA expression in liver from obese mice after 7 weeks of HFD administration, treated with MPEP 20 mg/Kg or vehicle; (**b**) Il1β mRNA expression in liver from obese mice after 7 weeks of HFD ad-ministration, treated with MPEP 20 mg/Kg or vehicle; (**c**) Tnf-α mRNA expression in liver from obese mice after 7 weeks of HFD ad-ministration, treated with MPEP 20 mg/Kg or vehicle; (**d**) Nrf2 mRNA expression in liver from obese mice after 7 weeks of HFD ad-ministration, treated with MPEP 20 mg/Kg or vehicle. Results are mean ± SE of 6 individual determinations.

The mRNA levels of common hepatic inflammatory markers such as interleukin-6 (Il-6), interleukin 1β (Il1β) and tumor necrosis factor-α (Tnf-α) were investigated (Figure 10a–c). All tested inflammation markers showed a significant reduction in their expression after MPEP administration (Il-6: *p* = 0.022; Il1β: *p* = 0.043; Tnf-α: *p* = 0.042). These new data are completely in accordance with the reduction of p-NFκB protein expression, confirming the role of MPEP in reducing inflammation.

The transcription factor nuclear factor, erythroid derived 2, similar to 2 (Nrf2), is considered an oxidative stress-sensitive gene and is used as a biological marker of oxidative stress [35]. In accordance with the reduction in inflammatory markers, a significant reduction (*p* = 0.01) in the mRNA expression of Nrf2 was found in MPEP-treated mice (Figure 10d), suggesting that the mGluR5 blockade produces anti-inflammatory effects.

### 2.4. MPEP Reduces Hepatic Stellate Cells (HSC) Activation

**Figure 11:**
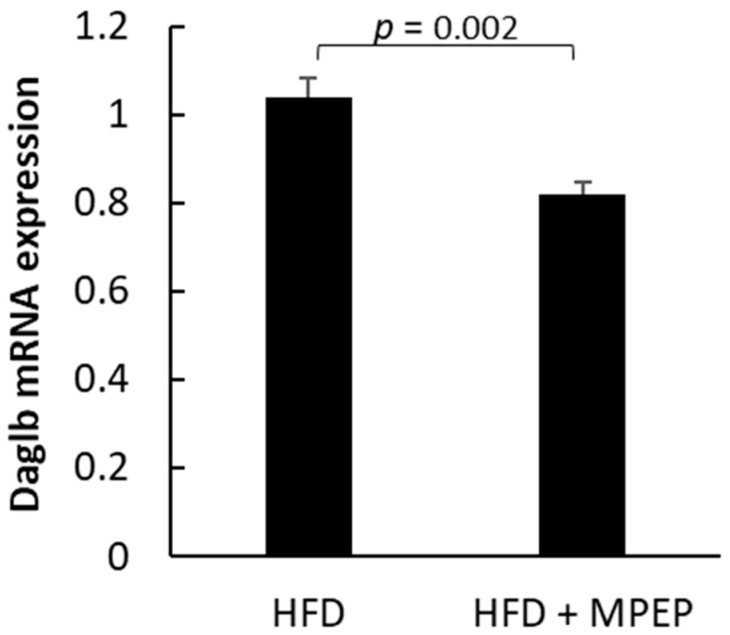
Hepatic stellate cell activation in obese mice. Daglb mRNA expression in liver from obese mice after 7 weeks of HFD administration, treated with MPEP 20 mg/Kg or vehicle. Results are mean ± SE of 6 individual determinations.

In a mouse model of alcoholic steatosis, mGluR5 mediated HSC activation, triggering the release of the endocannabinoid 2-arachidonoylglycerol (2-AG). 2-AG activates the hepatocyte cannabinoid receptor-1 (CB1R), which, in turn, promotes SREBP-1 activation in the hepatocytes, leading to de novo lipogenesis [22]. The enzyme diacylglycerol lipase-beta (Daglb) catalyzes the hydrolysis of arachidonic acid (AA)-esterified diacylglycerols (DAGs) in the principal endocannabinoid, 2-arachidonoylglycerol (2-AG), thus its expression could be a good indicator to evaluate the production of 2-AG. A significant reduction in Daglb mRNA expression in MPEP-treated mice was found (Figure 11; *p* = 0.002), indicating that the mGluR5 blockade reduces HSC activation.

**Figure 12:**
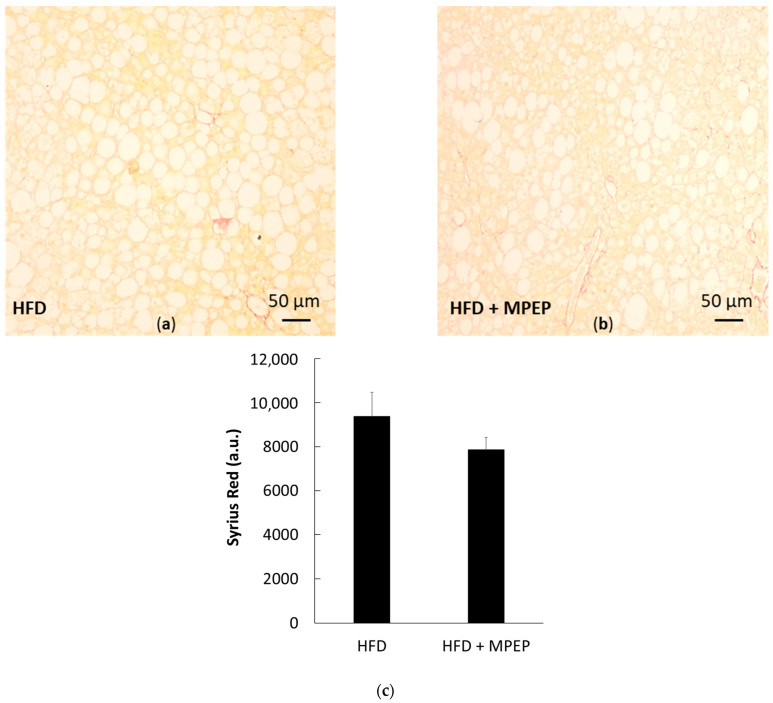
Sirius Red in obese mice. (**a**,**b**) Sirius red staining in liver sections from obese mice after 7 weeks of HFD administration treated with vehicle (**a**) or MPEP 20 mg/Kg (**b**); (**c**) Quantification of the Sirius red staining with ImageJ. HFD: high-fat diet + vehicle group; HFD + MPEP: high-fat diet + 20 mg/Kg MPEP group. Results are mean ± SE of 6 individual determinations.

Fibrosis was also evaluated in liver sections using the azo dye Sirius Red. The red dye was essentially visible in blood vessels and not in the parenchyma; no significant difference was found between the HFD and MPEP groups, showing that, in our 7-week model of NAFLD, the length of ROS exposure is not sufficient to induce collagen deposition (Figure 12a–c). In other works using the same animal model, significant levels of fibrosis were observed after at least a 12-week diet administration [24].

## 3. Discussion

De novo lipogenesis (DNL) is a highly regulated process converting carbohydrates from circulation into fatty acids, which are then used to synthesize triglycerides or exported as very low density lipoproteins (VLDL) [1]. Insulin resistance is considered to be one of the key factors involved in the increase in hepatic DNL and in the release of adipokines and inflammatory cytokines, leading to NAFLD and NASH [36,37]. The *ob/ob* mouse model is considered one of the most suitable models of metabolic syndrome; these mice develop NAFLD without spontaneously progressing to NASH [1,38,39,40] unless another insult is added, i.e., the administration of an HFD [24]. SREBP-1 is considered one of the pivotal transcription factors involved in the regulation of DNL [41]. In particular, SREBP-1C regulates the expression of genes that catalyze the synthesis of FAs, triglycerides (TGs), and NADPH required for FA synthesis. Both SREBP-1 mRNA and its active nuclear protein are increased in *ob/ob* mouse livers; in double mutant Lep*^ob/ob^* × Srebp-1^−/−^ mice, hepatic steatosis was markedly attenuated, but obesity and insulin resistance remained persistent [42]. More recent studies have been confirming that metabolic syndrome-driven NAFLD is closely related to lipid metabolism via SREBPs [26], which in turn are regulated by mTORC1 [26]. In this work, we found that *ob/ob* mice fed with an HFD for 7 weeks, and simultaneously administered with 20 mg/Kg MPEP displayed a decrease in body and liver weight and had a reduced fat liver accumulation and reduced lipid droplet area when compared with the vehicle group. In association with reduced fat accumulation, a major reduction in nuclear SREBP-1 was reported.

We also found that MPEP administration is associated with a significant increase in PPARα activation state, confirming our in vitro findings on HEPG2 cells [19]. PPARα, as well as SREBP-1, is considered a new relevant pharmacological target in NAFLD. In hepatocytes, PPARα participates in the regulation of genes involved in lipid metabolism, such as fatty acid binding protein (FABP)1, which controls free fatty acid (FFA) trafficking, delivery, and storage, or long and medium chain acyl-CoA dehydrogenases (LCAD and MCAD, respectively), which are involved in mitochondrial β-oxidation. A decrease in PPARα activation leads to the alteration of liver lipid homeostasis, lipotoxicity, and NASH [43]. In addition, PPARα negatively modulates inflammation by inhibiting the activity of pro-inflammatory transcription factors, such as NF-κB [44]. PPARα deletion has been found to impair fatty acid catabolism, resulting in hepatic lipid accumulation and liver inflammation in preclinical models of steatosis [44,45]. At the liver level, PPARα activation is mediated by N-methyl-D-aspartate receptor (NMDAR) inhibition, leading to the recovery of fatty acid oxidation and reduction of lipid accumulation in the liver of HFD-fed mice [46]. Since MPEP, in addition to its activity as a negative modulator of mGluR5, is capable of unspecific NMDAR inhibition [47], it is possible to hypothesize that in our model, MPEP was responsible for PPARα induction via the NMDAR receptor. In our model, MPEP-related PPARα overexpression may also contribute to SREBP-1 suppression. In fact, it was found in mouse primary hepatocytes that PPARα, by enhancing the insulin-induced gene (Insig)2a protein, suppresses SREBP-1c [48].

To further elucidate these molecular pathways, PPARα downstream genes Acox1 and Cpt1a, and of SREBP-1 downstream genes Acc1 and Fasn, were investigated. We found a significant increase in Acox1 mRNA expression after MPEP administration, while no differences in Cpt1a transcript were obtained between the two considered groups. The increase in Acox1 mRNA expression in the MPEP-treated group supports our hypothesis about the ability of MPEP to improve peroxisomal β-oxidation through PPARα activation. It is known that fatty acid oxidative PPARα target genes and PPARα expression are reduced in obese mice [49,50,51], as observed also in the present work in the untreated group. MPEP administration is able to almost double the expression of PPARα in our model of steatosis, and the increase in the mRNA expression of its target gene Acox1 confirm our hypothesis. In addition, PPARα not only regulates β-oxidation, but also suppresses the expression of lipogenic genes such as Acc1 [49]. Acc1 and Fasn are both target genes of SREBP-1, the lipogenic factor here investigated. The mRNA expression of the Acc1 gene was significantly decreased after MPEP administration, in accordance with the observed reduction of SREBP-1 protein expression. However, it is reasonable to think that the increased activity of PPARα may play an additional role in controlling lipogenesis via SREBP-1 suppression, as described by Konig et al. [52]. Regarding the mRNA expression of Fasn, no differences were found between the two groups; a possible explanation is that in obese mice, both SREBP-1 mRNA and its active nuclear protein expression are increased [42,53] and the period of MPEP treatment used in our experiments is not sufficient to downregulate both SREBP-1 and its target genes, while the combined activity of increased PPARα and decreased SREBP-1 is strong enough to observe a reduction in Acc1.

Despite these results, no reduction in phosphorylated mTOR was observed; thus, to clarify this point, we investigated any changes in the downstream mTOR effectors S6K1 and 4E-BP1 mRNA expression. We found a significant decrease in the mRNA expression of both considered genes in the MPEP-treated group. These data are apparently in contrast with the activation of mTORC1 in the MPEP-treated group shown in Figure 6a; however, literature data demonstrate that rapamycin insensitive regulation of S6K1 and 4E-BP1 can occur. S6K1 can be activated by other signaling pathways, such as the mitogen-activated protein kinase/extracellular signal-regulated kinase (MAPK/ERK) pathway, which can lead to the phosphorylation and activation of S6K1 in a manner that is independent of mTOR. Richards and colleagues demonstrated that the activation of S6K1 by the MAPK/ERK pathway requires the phosphorylation and activation of RSK1, a downstream effector of ERK, which in turn phosphorylates and activates S6K1 [29]. As for 4E-BP1, a mTOR independent regulation of this factor occurs in glioma cells [30], myoblasts [31], and acute myeloid leukemia [32], suggesting that 4E-BP1 activation could be regulated directly or indirectly by PI3K but not via Akt-mTORC1 [54]. This situation is in line with the reduction of S6K1 and 4E-BP1 mRNA expression found in the MPEP-treated group. Our work is only a first attempt to try to explain the possible role of MPEP in liver steatosis, and further studies are required to understand in depth the underling mechanisms in this process, but the hypothesis that MPEP inhibits the mTOR downstream effectors S6K1 and 4E-BP1 may explain in part the increased mTOR activation in the MPEP-treated group; mTORC1 regulates many cellular pathways and trying to blunt its regulatory activity can lead to unexpected feedback loops [55]. The activation of mTORC1 in our MPEP-treated group could be a positive feedback to counteract the possible MPEP-mediated inhibition of S6K1 and 4E-BP1. In addition, mTORC1 has been studied for a long time as a possible target to control lipid homeostasis in the liver, but to the best of our knowledge, its role is still unclear, and several studies show conflicting results regarding the role of mTOR in NAFLD [55]. For example, Peterson and colleagues showed that the deletion of the regulatory-associated protein of mTOR (Raptor) and consequent mTOR inactivation lead to the inhibition of de novo lipogenesis mediated by SREBP-1 in hepatocytes [56]. On the contrary, other studies demonstrated that activating mTORC1 with the deletion of tuberous sclerosis 1/2 (TSC 1/2) complex, which is a negative regulator of mTORC1, protected mice from liver steatosis [57], while other groups observed increased steatosis and liver injury in mice deficient for Raptor in the liver [58]. Thus, the real role of mTORC1 in NAFLD is still poorly understood.

Lastly, an mTOR-independent increase in SREBP-1 was previously observed in a work by Choi et al. (2019). In this work, it has been demonstrated that mGluR5 mediates HSC activation in a murine model of alcoholic steatosis, leading to the release of the endocannabinoid 2-arachidonoylglycerol (2-AG). 2-AG activates the hepatocyte cannabinoid receptor-1 (CB1R), which, in turn, triggers SREBP-1 activation and translocation to the nucleus of hepatocytes, leading to triglyceride and cholesterol synthesis through its target genes [22]. The described mechanism is reasonably responsible for SREBP-1 modulation also in the model of non-alcoholic steatosis here adopted; in fact, the distinction between alcoholic and non-alcoholic disease is currently being questioned by experts also on the basis of shared mechanisms concurring to fat accumulation [59]. In this regard, the obtained result about the mRNA expression reduction of diacylglycerol lipase-beta (Daglb) in MPEP-treated group corroborates this speculation. In fact, Daglb catalyzes the hydrolysis of arachidonic acid (AA)-esterified diacylglycerols (DAGs) to produce the principal endocannabinoid, 2-AG, thus its expression could be a good indicator to evaluate the production of 2-AG and, consequently, to observe HSC activation. However, we previously demonstrated that an HSC-independent, mGluR5-mediated, fat accumulation is also possible in an hepatocyte-only in vitro model, then without any intervention of HSCs: in that work, we showed that the blockade of the mGluR5 receptor with MPEP leads to a reduction in both fat accumulation and SREBP-1 expression in HEPG2 cells treated with oleate and palmitate [19]. In this in vivo model, both mechanisms, HSC-dependent and independent, are probably involved in the modulation of liver lipogenesis. 

The NF-κB signaling pathway is believed to regulate inflammation in NASH progression, even though this relationship is still unclear. During NASH progression, mitochondria are compromised by excessive β-oxidation, which leads to mitochondrial dysfunction and oxidative stress triggered by several signaling pathways involving, as previously demonstrated, the activation of the NF-κB. In fact, NF-κB induces ROS production via transcriptional regulation of oxidant genes and the regulation of the inflammasome-proinflammatory cytokine axis in macrophages [60]. However, the role of NF-κB may not be limited to the regulation of oxidative stress; in fact, in the liver of mice, a high carbohydrate diet induced an enforced nuclear shuttling of hepatic NF-κB, in association with an increased DNL [61]. Here and in our previous in vitro data, we found that the blockade of the mGluR5 receptor leads to a reduced nuclear translocation of NF-κB, in association with a reduction in oxidative stress. The relationship between the mGluR5 receptor and NF-κB has been previously investigated in tissues other than the liver. In the hippocampus of mice, the activation of mGluR5 was found to positively regulate NF-κB activity by means of a time-dependent increase in the DNA binding activities of p50, p65, and c-Rel; conversely, MPEP inhibited the activation of NF-κB [62]. The blockade of mGluR5 also protected astrocytes from metamphetamine-mediated increases in NF-κB activation [63]. mGluR5 agonists also amplified irradiation-induced NF-κB activation in T-cells [64]. This relationship has not been demonstrated in the liver yet, and with the findings here presented, we can confirm an association between mGluR5 inhibition and a decrease in NF-κB nuclear translocation and activation. This finding is in line with the results here presented about the reduced levels of common hepatic inflammatory markers such as interleukin-6 (Il-6), interleukin 1β (Il1β) and tumor necrosis factor-α (Tnf-α) in the MPEP-treated group. In addition, in accordance with the reduction in inflammatory markers, such as interleukins and p-NFκB, we observed a significant reduction in the mRNA expression of Nrf2 in the MPEP-treated mice. Nrf2 is a transcription factor, which plays a pivotal role in controlling the expression of many antioxidant genes, such as NAD(P)H quinone oxidoreductase (NQO), and exerting anti-inflammatory and anti-oxidant functions [65]; Nrf2 is also considered an oxidative stress-sensitive gene and is used as a biological marker of oxidative stress [35]. This result confirms once again that the administration of MPEP produces anti-inflammatory effects in this model of hepatic steatosis.

In this 7-week model of NAFLD, we observed an increase in ROS production and oxidative stress injury and a significant MPEP-induced protective effect, confirming previous results in different models of inflammation [14,15,16,17,18,66]. However, this insult did not result in the development of a significant level of fibrosis, and consequently, we were not able to observe a significant protective effect of MPEP in terms of a reduction in collagen deposition. This finding was expected; in fact, it was demonstrated in previous works that 12–18 weeks of HFD administration are necessary for fibrosis development [24].

Concluding, here we show for the first time that mGluR5 blockade induces a reduction of lipid accumulation and oxidative stress in *ob/ob* mice fed with an HFD for 7 weeks. The MPEP-induced reduction of lipid accumulation is associated with an mTOR-independent strong suppression of SREBP-1 activity and an increase in PPARα, possibly responsible for the modulation of lipogenesis; MPEP administration also resulted in a reduction in NF-κB activation, responsible for the lowered oxidative stress. These data consolidate the notion that hepatic mGluR5 has a relevant role in NAFLD development and encourage further studies to better understand the interconnection between this receptor and the multiple nuclear factors involved in the pathogenesis of this disease.

## 4. Materials and Methods

### 4.1. Animals and Experimental Model

The animal model used was approved by the Italian Ministry of Health and the Pavia University Animal Care Commission. 4-week old male B6.Cg-Lepob/J (*ob/ob*) mice (Charles River, Calco, LC, Italy) were fed a high-fat diet for 7 weeks. After a 1-week quarantine period, HFD administration started simultaneously with IP daily administration of 20 mg/Kg MPEP (*n* = 6) or vehicle (Natrosol, *n* = 6). After 7 weeks, mice were anesthetized (Pentobarbital IP injection, 40 mg/Kg), blood was collected from the inferior caval vein, the livers were exposed, and the tissue specimens were collected from the medium lobe, immediately frozen in liquid nitrogen until being processed for biochemical assays, or fixed in formalin for paraplast inclusion.

### 4.2. Materials

MPEP was purchased from Tocris Cookson (Bristol, UK). The high-fat diet “D09100301” (Research Diets, Inc., New Brunswick, NJ, USA) was provided by Laboratorio Dottori Piccioni (Gessate, MI, Italy). All other chemicals, when not specified, were purchased from Sigma-Aldrich (Milano, Italy).

### 4.3. Tissue Histology and Staining

Fresh liver samples from mice were fixed in 2% p-formaldehyde in 0.1 M phosphate buffer at pH 7.4 for 24 h and processed routinely until they were embedded in Paraplast wax. Samples were sliced into 8-μm-thick sections and stained with Hematoxylin–Eosin (Sakura Finetek, Mestre, Italy) or Sirius Red (Direct Red 80, Sigma-Aldrich, Milano, Italy). Slices were then evaluated under a light microscope (Nikon Eclipse E800, Nikon Europe, Amstelveen, The Netherlands). Lipid droplet area and parenchymal fibrosis were estimated using ImageJ. 

### 4.4. Blood Sample Preparation and Plasma Cholesterol and Triglycerides Evaluation

Blood samples were obtained after heparin injection (10,000 U/mL in PBS, heparin, sodium salt, porcine intestinal mucosa, Sigma-Aldrich, Milano, Italy) in the vena cava. After collection, blood samples were centrifuged at 3000 rpm for 15 min, RT to obtain plasma. Aliquots of plasma samples were sent to MyLav–Laboratorio La Vallonea (Passirana di Rho, Italy) for cholesterol and triglyceride content evaluation.

### 4.5. Hepatic Lipid Extraction and Quantification

Hepatic lipid extraction was performed according to Lyn-Cook et al. [67]. Frozen tissues (50–70 mg each) were homogenized in 200 µL of water. Lipids were extracted by adding 1 mL chloroform-methanol (2:1), and samples were incubated for 1 h at room temperature with intermittent agitation. After centrifuging at 3000 rpm for 5 min at room temperature, the separated lipid-containing lower fraction was transferred to a clean tube and N_2_-dried. Pellets were re-suspended in 100 µL of 100% ethanol. 12.5 µL aliquots of lipid extract were added to 475 µL of phosphate-buffered saline (PBS) in a UV quartz cuvette, and then, 12.5 µL of Nile red solution (1 mg/mL in DMSO) was added. Fluorescence intensity (Ex 485/Em 572) was measured using a fluorescence spectrometer LS 50 B (Perkin-Elmer Inc., Waltham, MA, USA). Results are expressed as total lipid/liver weight (mg/g).

### 4.6. Analysis of Oxidative Stress and Radical Oxygen Species

Tissue thiobarbituric acid reactive substances (TBARS, nmol/mg prot) were used as an index of lipid peroxidation; TBARS concentration in liver homogenates was measured according to the method of Esterbauer and Cheeseman [68]. The TBARS concentrations were calculated using malondialdehyde (MDA) as a standard [69]. ROS (A.U.) were quantified on liver homogenates using the DCFH-DA method, based on the ROS-dependent oxidation of DCFH to DCF. Protein content was assayed by the method of Lowry et al. [70].

### 4.7. Nuclear Extracts

Mouse liver nuclei were isolated according to Nagata et al. [71]. After injection of heparin (10,000 U/mL in PBS) in the vena cava, liver was excised, and the left and right lobes were homogenized in 4 mL of ice-cold Buffer A (250 mM sucrose, 5 mM MgCl_2_, and 10 mM Tris–HCl [pH 7.4]) per 0.5 g of the liver. The homogenate was centrifuged at 600× *g* for 10 min at 4 °C, and the obtained pellet was resuspended in 14 mL of ice–cold Buffer A. After a second centrifuge at 600× *g* for 10 min at 4 °C, the nuclei pellet was resuspended in 9 volumes of ice-cold Buffer B (2.0 M sucrose, 1 mM MgCl_2_, and 10 mM Tris–HCl [pH 7.4]) and then centrifuged at 16,000× *g* for 30 min at 4 °C. The white nuclei pellet at the bottom of the tube was separated from the brownish and sticky one in the upper part of the tube, resuspended in ice-cold Buffer A, and snap-frozen in liquid nitrogen [71].

### 4.8. Western Blot

Liver tissue samples were homogenized in an ice-cold lysis buffer supplemented with protease inhibitors and centrifuged at 15,000× *g* for 10 min. Samples of liver or nuclear extracts containing the same number of proteins were separated in SDS-PAGE on 7.5% acrylamide gels and transferred to PVDF membrane. Unspecific sites were blocked for 2 h with 5% Bovine Serum Albumin (BSA) in TBS Tween (20 mM Tris/HCl, 500 mM NaCl, pH 7.5, 0.1% Tween 20) at 4 °C. The membranes were incubated with primary antibodies overnight at 4 °C, under gentle agitation. 

Primary antibodies against mouse monoclonal PPARα, NF-κB, phospho-NF-κB, HDAC, alpha tubulin (DM1A), and vinculin were used at 1:1000 dilution, while a mouse monoclonal antibody against SREBP-1 was used at 1:500 dilution. Rabbit monoclonal anti-mTOR and phospho-mTOR were used at 1:1000. Membranes were washed in TBS Tween (Na_2_HPO_4_ 8 mM, NaH_2_PO_4_-H_2_O_2_ mM, NaCl 140 mM, pH 7.4, 0.1% Tween 20) and incubated with peroxidase-conjugated secondary anti-rabbit or anti-mouse antibodies at a 1:1000 dilution for PPARα, SREBP-1, mTOR, phospho-mTOR (Ser2448), HDAC, and NF-κB; the dilution 1:2000 was used for phospho-NF-κB and tubulin, while a 1:3000 dilution was used for vinculin. PPARα, SREBP-1, NF-κB, phospho-NF-κB, and vinculin were bought from Santa Cruz Biotechnology. Rabbit monoclonal anti-mTOR and phospho-mTOR and mouse monoclonal anti-HDAC were purchased from Cell Signaling Technology (Danvers, MA, USA), while mouse monoclonal anti-tubulin was from Sigma-Aldrich (Milano, Italy). Immunostaining was revealed with BIO-RAD Chemidoc XRS+ visualized using the ECL Clarity BIO-RAD (Segrate, MI, Italy). Band intensity quantification was performed by BIO-RAD Image Lab Software™6.0.1, (Segrate, MI, Italy).

### 4.9. RT-PCR Analysis

A median lobe of frozen livers was used for total RNA extraction in TRI reagent (Sigma-Aldrich, Milano, Italy) according to the Chomczynski method [72]. Total RNA was quantified by measuring the absorbance at 260 nm with a T92+ UV Spectrophotometer, and the purity degree was evaluated using the ratio between the absorbances 260/280 nm. The c-DNA was generated using iScript Supermix (BIO-RAD, Segrate, MI, Italy) as previously described [18]. The qPCR reactions were performed using the CFX96TM Real-Time System (BIO-RAD, Segrate, MI, Italy) and by mixing 10 μL of SsoAdvancedTM SYBR^®^ Green Supermix (BIO-RAD, Segrate, MI, Italy), 1 μL of the oligonucleotide primer (10 pmol/μL), and 2 μL of cDNA (2.5 ng/μL) to reach a final volume of 20 μL/well. Amplification was performed through two-step cycling (95–60 °C) for 40 cycles, following the supplier’s instructions. All samples were assayed in triplicate. Acox1, Cpt1a, Acc1, Fasn, S6K1, 4E-BP1, Il-6, Il1β, Tnf-α, Nrf2, Daglb, Ubc, Gapdh, and Rps9 gene amplification efficiencies were established by means of calibration curves (95.7%, 93.7%, 94.6%, 88.8%, 93.3%, 93.6%, 96%, 107.4%, 100.3%, 94%, 82.8%, 124.7%, 111.7%, and 112.2%, respectively) in a cDNA concentration range of 5–0.625 ng/μλ. The expression of the three reference genes remains constant in the two experimental groups. The amplicon context sequence of the primers (PrimePCR, BIO-RAD, Segrate, MI, Italy) Acox1 (unique assay ID: qMmuCID0016828), Cpt1a (unique assay ID: qMmuCID0021095), Acc1 (unique assay ID: qMmuCID0006041), Fasn (unique assay ID: qMmuCED0045676), S6K1 (unique assay ID: qMmuCID0022303), 4E-BP1 (unique assay ID: qMmuCID0006526), Il-6 (unique assay ID: qMmuCID0005613), Il1β (unique assay ID: qMmuCID0005641), Tnf-α (unique assay ID: qMmuCED0004141), Nrf2 (unique assay ID: qMmuCID0021433), Daglb (unique assay ID: qMmuCID0005918), Ubc (unique assay ID: qMmuCID0021036), Gapdh (unique assay ID: qMmuCED0027497) and Rps9 (unique assay ID: qMmuCED0037603) are displayed in Table 2, which lists the amplicon sequences with additional base pairs added to the beginning and/or end of the sequence. This is in accordance with the minimum information for the publication of real-time quantitative PCR experiments (MIQE) guidelines, as reported by Bustin et al. [73]. Gene expression was calculated using the ΔCt method. The comparison between the two groups was calculated using the ΔΔCt method.

### 4.10. Statistical Analysis

All data were statistically analyzed using R statistical software (version 4.1.0) and RStudio integrated development environment (version 2022.02.3 Build 492).

Values are presented as the average ± SEM. Statistical analysis was performed with the Student’s T Test. When data distribution was not normal, Wilcoxon’s Rank Test was used. The value of *p* < 0.05 was used as the level of significance.

## Figures and Tables

**Table 1:** Model characterization and liver injury evaluation.

	HFD	HFD + MPEP
Body weight (g)	46 ± 0.97	40.83 ± 1.51 ***
Liver weight (g)	5.37 ± 0.32	4.26 ± 0.17 ***
AST (mg/dL)	537 ± 77.93	520.33 ± 30.67
ALT (mg/dL)	523.2 ± 49.47	485.6 ± 17.07
Glucose (mg/dL)	196.5 ± 35.22	199.33 ± 32.59

*** *p* ≤ 0.01. Results are mean ± SE of 6 individual determinations.

**Table 2:** Amplicon sequences of primers used in this study. Acetyl-Coenzyme A carboxylase alpha (Acc1 or Acaca); acyl-Coenzyme A oxidase 1, palmitoyl (Acox1); carnitine palmitoyltransferase 1a, (liver isoform, Cpt1a); diacylglycerol lipase, beta (Daglb); eukaryotic translation initiation factor 4E binding protein 1 (4E-BP1 or Eif4ebp1); fatty acid synthase (Fasn); interleukin 1 beta (Il1β); interleukin 6 (Il-6); nuclear factor (erythroid-derived 2)-like 2 (Nrf2 or Nfe2l2); ribosomal protein S6 kinase, polypeptide 1 (S6K1 or Rps6kb1) and tumor necrosis factor alpha (Tnf-α) were the genes of interest to be analyzed. Glyceraldehyde-3-phosphate dehydrogenase (Gapdh), ubiquitin c (Ubc), and ribosomal protein S9 (Rps9) were used as reference genes.

Gene	Amplicon Sequence
*Acc1* *(Acaca)*	GAGAAGGAGGGCTCCCTGTCACCAGCCTCCGTCAGCTCAGATACACTTTCTGAT TTGGGGATCTCTGGCTTACAGGATGGTTTGGCCTTTCACATGAGATCCAGCATGT CTGGCTTGCACCTAGTAA
*Acox1*	TACGACACCATACCACCCACCAGCTTCCCCGACTGAACCTGGTCATAGATTTTCA TCAAGAACCTGGCCGTCTGCAGCATCATAACAGTGTTCTCCC
*Cpt1a*	TACATCTACCTGCGGGGCCGAGGGCCGATCATGGTTAACAGCAACTACTACGCC ATGGAGATGCTCTACATCACCCCAACCCATATTCAGGCAGCGAGAGCTGGCAAC ACCATCCACGCCATACTGC
*Daglb*	GGAGGACCTCAAGAGGAGGATCCTGAGAGTGATCGCTAACTGCAATAAGCCGAA GTACAAGATCTTGCTGCATGGCTGTTGGTACGGACTGTTCGGAGGAAGCCCTG
*4E-BP1* *(Eif4ebp1)*	GAACCAGGATTATCTATGACCGGAAATTTCTGATGGAGTGTCGGAACTCACCTGT GGCCAAAACACCCCCAAAGGACCTGCCAGCCATTCCTGGGGTCACTAGCCCTAC CAGCGATGAGCCTCCCATGCAAGCCAGCCAGAGCCAACTGCCCAGCAGCCCGG AAGATAAGCGGGCAGGCGGTGAAGAGTCACAATTTGAGATGGACATTTAAGGGA CCAGCCGTAGGAC
*Fasn*	ACTAGAGCCAACCAGATGCTTCAGTCTTAGGCTAATCACTCAGACTGGGATCCTC TGAACACCTTTGTACCCTACCAATGCAGTTGTCCTCTGGATGCTCCTCTCTGGAT GTGATCGAATGCTGCG
*Gapdh*	TGGGAGTTGCTGTTGAAGTCGCAGGAGACAACCTGGTCCTCAGTGTAGCCCAAG ATGCCCTTCAGTGGGCCCTCAGATGCCTGCTTCACCACCTTCTTGATGTCA
*Il1β*	TATTTTGTCGTTGCTTGGTTCTCCTTGTACAAAGCTCATGGAGAATATCACTTGTT GGTTGATATTCTGTCCATTGAGGTGGAGAGCTTTCAGCTCATATGGGTCC
*Il-6*	ACAAGAAAGACAAAGCCAGAGTCCTTCAGAGAGATACAGAAACTCTAATTCATAT CTTCAACCAAGAGGTAAAAGATTTACATAAAAT
*Nrf2* *(Nfe2l2)*	ACTTACTCCAAGATCTATGTCTTGCCTCCAAAGGATGTCAATCAAATCCATGTCCT GCTGGGACTGTAGTCCTGGCGGTGGCAACTCCAAGTCCATCATGCTGAGGGCG GACGCTGTGGTAGGGC
*Rps9*	CGTCCAGGCCGAGTGAAGAGGAAGAATGCCAAGAAAGGCCAGGGCGGGGCTG GAGCTGGTGATGATGAGGAAGAGGATTAATTAATACTTGGCTGAACTGGAGGAT TGTCTAGTTTTCC
*S6K1* *(Rps6kb1)*	TTTTTAAGCACCTTCATGGCAAATATCTTCCCAGTATTTGCTCCTGTTACTTTTCG TACTTGAAAAACCTTTCCATAGCCCCCTTTACCAAGTACCCGAAGTAGCTCAAAA CATTCTGGTCTGATTTTTTCTGGCCCTCTGTTCACACTAGTTTCTGAGATTTCAAA TTTCTCACAATGTTCCATGCCAAGTTCATATGGTCCAACTCCC
*Tnf-α*	CCACAAGCAGGAATGAGAAGAGGCTGAGACATAGGCACCGCCTGGAGTTCTGG AAGCCCCCCATCTTTTGGGGGAGTGCCTCTTCTGCCAGTTCCACGTCGCGGATC ATGCTTTCTGTGCTCATGGTGTCTTTTCTGGAGGGAGATGTG
*Ubc*	GATGCCCTCCTTGTCCTGGATCTTTGCCTTGACATTCTCAATGGTGTCACTGGGC TCGACCTCCAGGGTGATGGTCTTACCAGTTAAGGTTTTCACAAAGATCTGCATCG TCTCTCTCACGGAGTTGTTTCACGGTGGCGTCCAGA

## Data Availability

The data presented in this study are available on request from the corresponding author.
